# Supplementary material for: Genetic Analysis for the Flag Leaf Heterosis of a Super-Hybrid Rice WFYT025 Combination Using RNA-Seq
Source: Plants (Basel). 2023 Jun 29;12(13):2496. doi: 10.3390/plants12132496 (PMC10346679; doi:10.3390/plants12132496)

Supplementary data

Supplementary Figures and Tables Captions

Supplementary Table S1. Summary of reads quality control.

Sample	Raw Data Read	Valid Data Read	Q20%	Q30%
WFB1YE1	58067824	51827564	99.97	96.90
WFB1YE2	55856602	50440052	99.97	96.42
WFB1YE3	49093548	44323472	99.97	96.93
WFB10YE1	55154582	48373938	99.97	96.89
WFB10YE2	32577058	29566054	99.97	97.09
WFB10YE3	57707296	53939666	99.97	96.82
CH1YE1	47223226	43972676	99.97	96.90
CH1YE2	50148850	47276426	99.97	96.38
CH1YE3	59521130	56657380	99.97	96.43
CH10YE1	49924152	47693980	99.96	95.71
CH10YE2	56697348	53102578	99.96	96.23
CH10YE3	51910576	49064482	99.96	96.10
WFY1YE1	42521008	39987756	99.97	96.29
WFY1YE2	42407886	38573182	99.97	95.61
WFY1YE3	49399744	44670540	99.97	95.66
WFY10YE1	59642288	54054670	99.96	96.48
WFY10YE2	58586170	53489318	99.96	96.37
WFY10YE3	59388316	57894440	99.95	95.66
WFB1YM1	41877940	37466360	99.96	95.28
WFB1YM2	37675788	34596514	99.96	95.32
WFB1YM3	43931940	40412444	99.96	95.25
WFB10YM1	55179068	48185880	99.96	96.55
WFB10YM2	37529196	33435912	99.97	96.20
WFB10YM3	36929212	32208332	99.96	95.35
CH1YM1	37453408	31809752	99.96	95.95
CH1YM2	49902748	44269166	99.96	96.03
CH1YM3	39974474	34311360	99.96	95.72
CH10YM1	42010238	36958032	99.97	95.77
CH10YM2	48782678	43370946	99.96	96.03
CH10YM3	49215802	43682886	99.96	96.08
WFY1YM1	58915542	52942812	99.96	96.32
WFY1YM2	35801982	31728748	99.96	95.16
WFY1YM3	38811202	32672228	99.96	95.73
WFY10YM1	45758222	39934752	99.96	95.85
WFY10YM2	49112104	40312014	99.96	95.47
WFY10YM3	41302046	36012804	99.96	95.36

CH, WFY and WFB represent CHT025, WFYT025 and WFB, respectively. E and M represent early and middle.Y represent flag leaf.1,10 represent 1 DPA and 10 DPA. For example, WFB1YE1 represent in the early rice environment, the flag leaf of WFB at 1 DPA.

Supplementary Table S2. Primers used in this study.

Gene ID	Forward primer	Reverse primer
OS09G0555500	GCAATCTGGGCGATATACGTGTG	TGTACCTCGACTTCGTTAGGTC
OS04G0578400	TGCCGATGACCGAGATGTTT	CATGCCGAACAGCGTAATCC
OS03G0592500	TATGGACCTGACCGTCCCAA	CTTCACCCCGTTCTTGGACA
OS03G0856700	CGGCTTCTTCCTGGTGGTTA	GTCGTTGCGCTGGAAGAATC
OS04G0611800	TACAAGAGGATTCGGCGTGG	CATCCCCATGGCAAGGCTAA
OS01G0580500	GGAGGTGAAGAGCTTCGTGT	AGTAGGTGGCCTCCCAGTC
OS01G0656200	CAGCAGGTGGCAGGGTTATC	GATACCACATCCCACAGCCC
OS01G0841600	CCTTCACTGGCGAGATCAGT	GGTCTCACCAATGCAAGCAA
OS02G0171100	TCGAGTGGCGAGAAGACAAA	CCCCAAGGAATATCTGCTGGA
Os11g0171300	TCCTTGCCATGGATGAGTCG	GAGACCTTGGCACCATGACT
Os06g0133800	GACGAGGTCATGCGGTACAA	GATGGCCTGGAGTCTTGCTT
Os08g0434300	AATTGCTGCCGAGGTTCTGA	CATGACCACCGACAACTGGA

Supplementary Figure S1 Comparison of the log2 (FC) of 12 randomly selected transcripts using RNA-Seq and qRT-PCR. **A-C**: In the early rice environment, compare 1 DPA and 10 DPA about CHT025, WFB and WFYT025, respectively. **D-E**: In the middle rice environment, compare 1 DPA and 10 DPA about CHT025, WFB and WFYT025, respectively.

A.

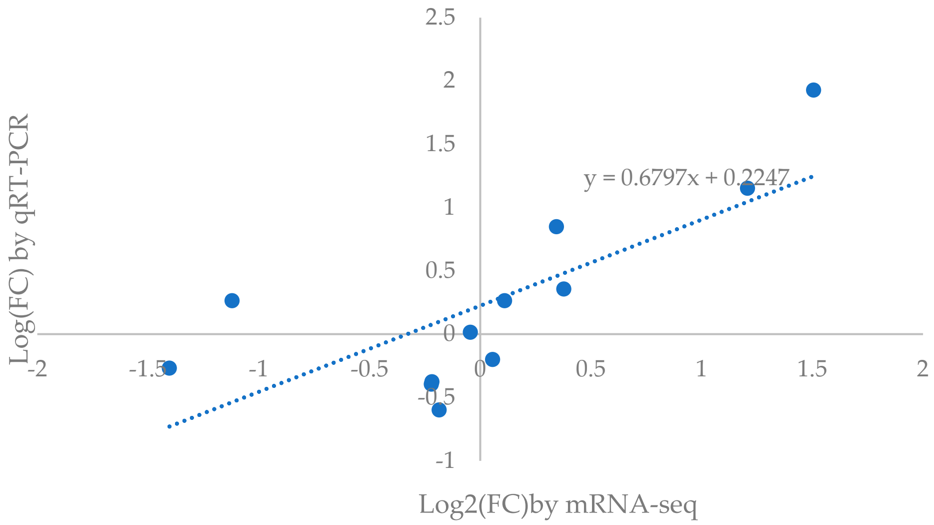

2.5
2
1.5
1
0.5
0
-0.5
-1
-2
-1.5
-1
-0.5
0
0.5
1
1.5
2
y = 0.6797x + 0.2247
Log(FC) by qRT-PCR
Log2(FC)by mRNA-seq

B

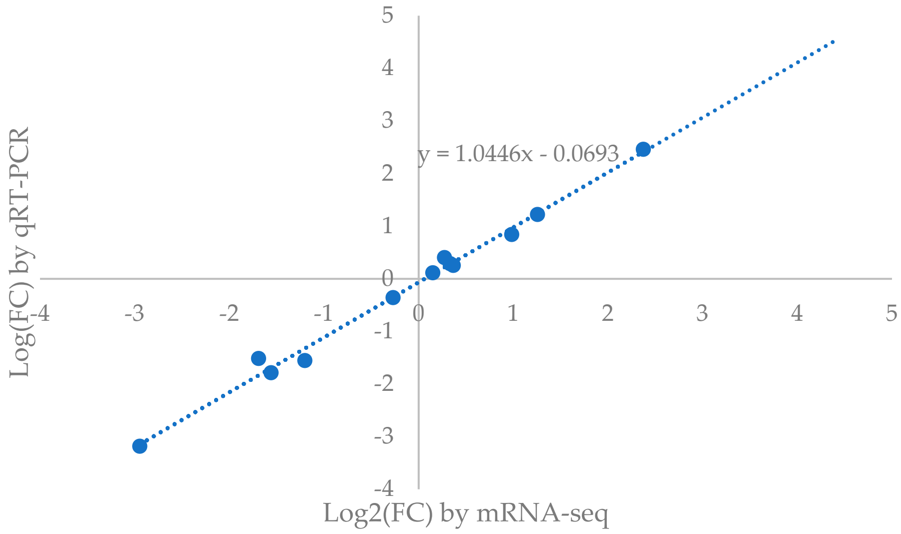

5
4
3
2
1
0
-1
-2
-3
-4
-4
-3
-2
-1
0
1
2
3
4
5
y = 1.0446x - 0.0693
Log(FC) by qRT-PCR
Log2(FC) by mRNA-seq

C

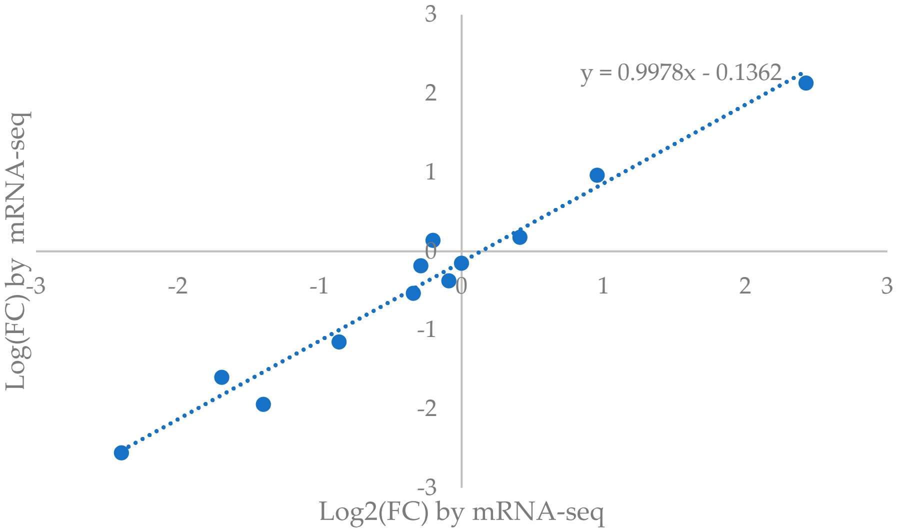
y = 0.9978x - 0.1362
Log(FC) by mRNA-seq
Log2(FC) by mRNA-seq
3
2
1
0
-1
-2
-3
-3
-2
-1
0
1
2
3

D

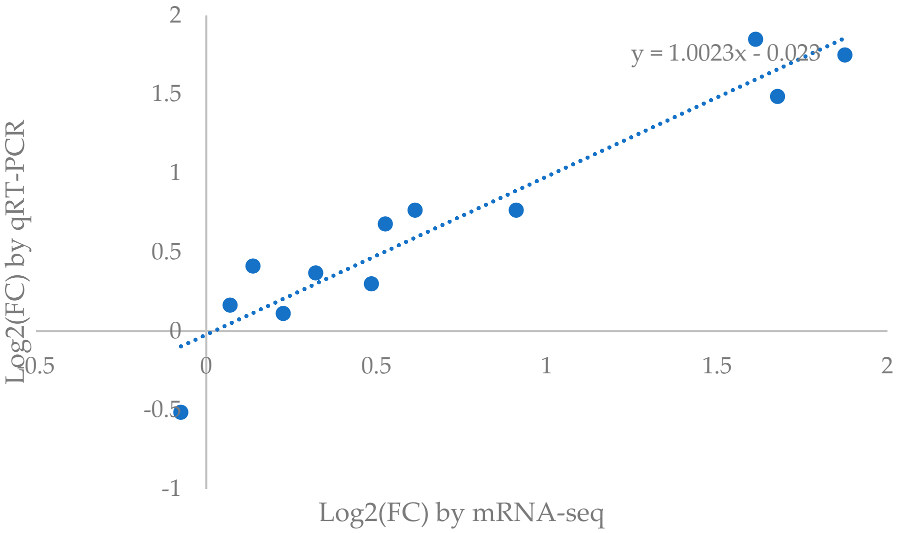
y = 1.0023x - 0.023
Log2(FC) by qRT-PCR
Log2(FC) by mRNA-seq
2
1.5
1
0.5
0
-0.5
-1
-0.5
0
0.5
1
1.5
2

E

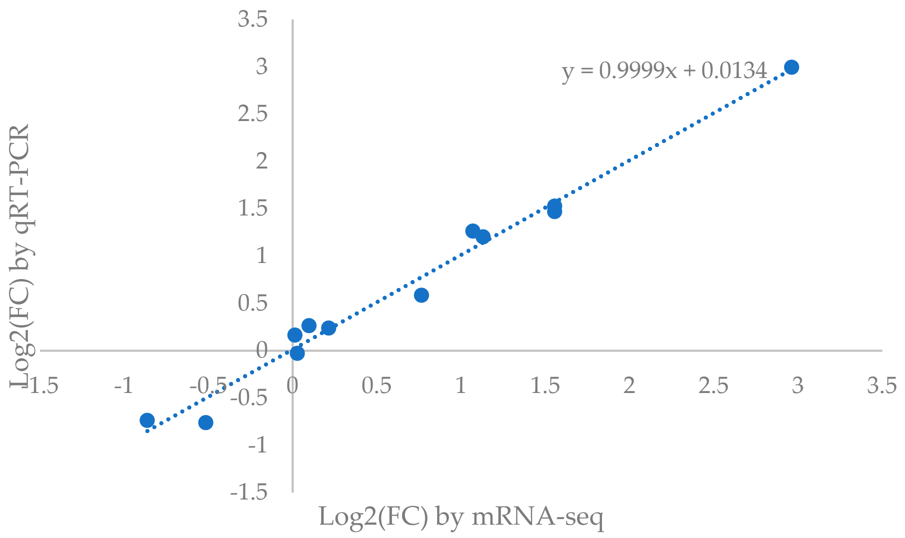

F

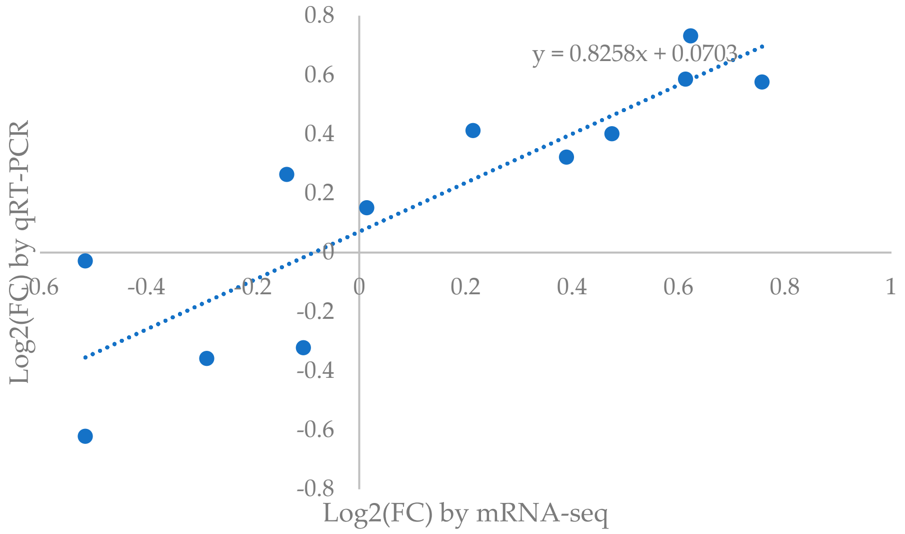

Supplementary Figure S2 Gene ontology (GO) enrichment analysis of DEGs**(A)** GO enrichment analysis of DEGs at 1 DPA and 10 DPA under the environment of early rice in WFYT025. **(B)** GO enrichment analysis of DEGs at 1 DPA and 10 DPA under the environment of middle rice in CHT025. **(C)** GO enrichment analysis of DEGs at 1 DPA and 10 DPA under the environment of middle rice in WFB. **(D)** GO enrichment analysis of DEGs at 1 DPA and 10 DPA under the

environment of middle rice in WFYT025.

A.

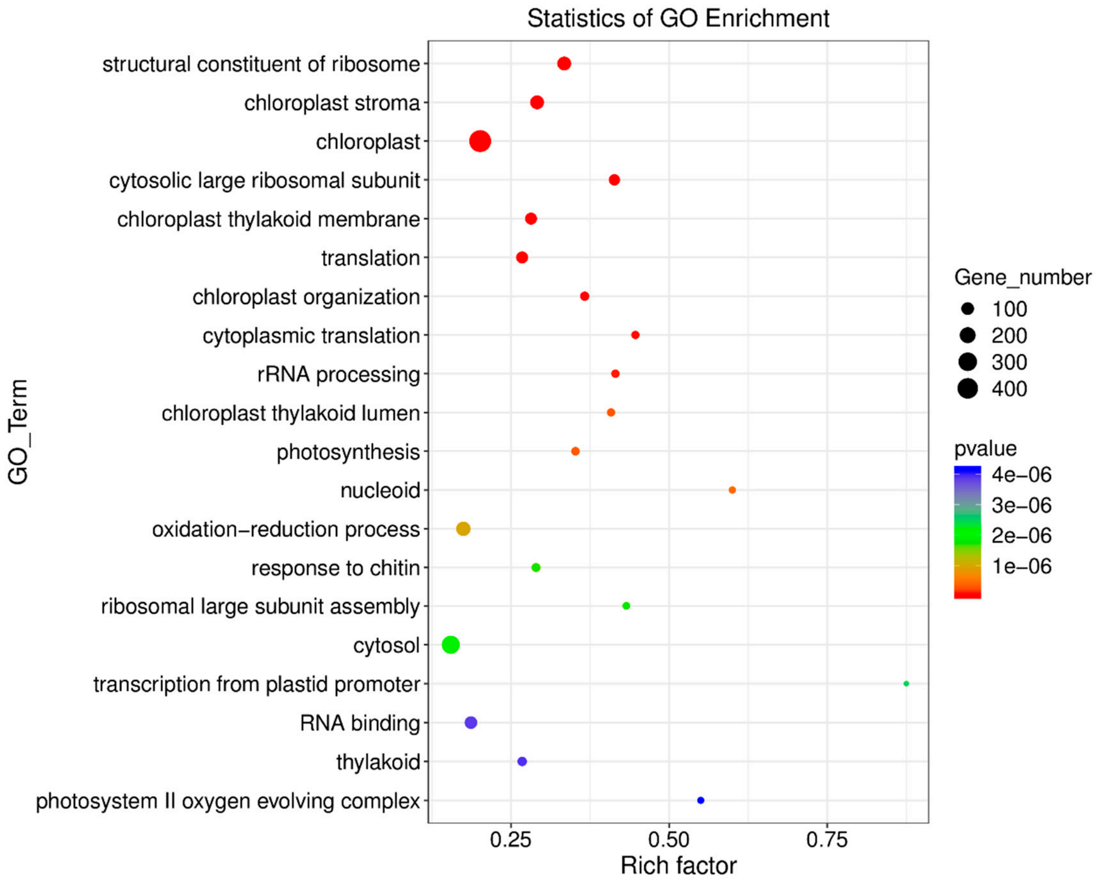

B

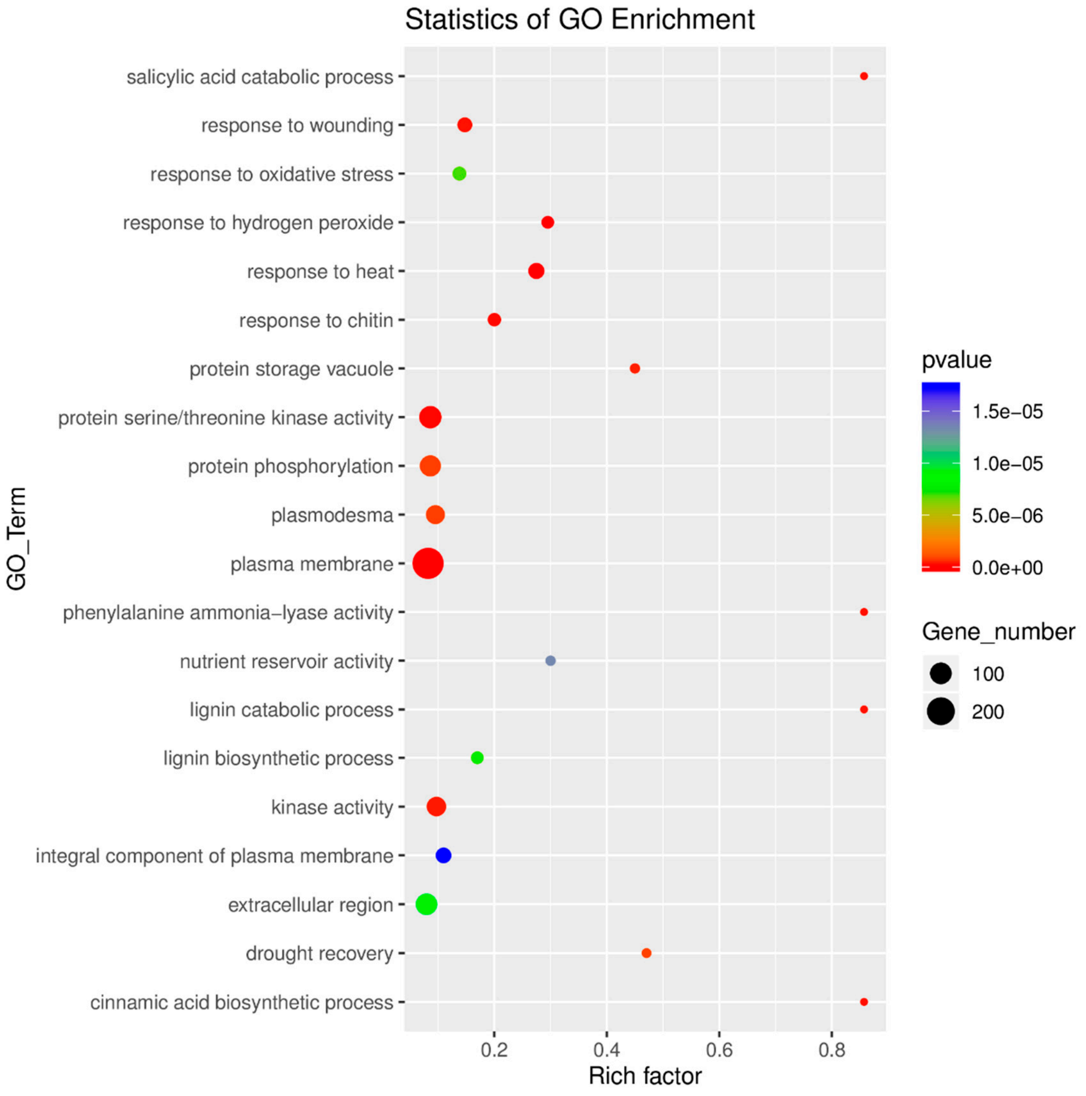
Statistics of GO Enrichment
salicylic acid catabolic process
response to wounding
response to oxidative stress
response to hydrogen peroxide
response to heat
response to chitin
protein storage vacuole
protein serine/threonine kinase activity
protein phosphorylation
plasmodesma
plasma membrane
phenylalanine ammonia–lyase activity
nutrient reservoir activity
lignin catabolic process
lignin biosynthetic process
kinase activity
integral component of plasma membrane
extracellular region
drought recovery
cinnamic acid biosynthetic process
GO_Term
0.2
0.4
0.6
0.8
Rich factor
pvalue
1.5e−05
1.0e−05
5.0e−06
0.0e+00
Gene_number
100
200

C

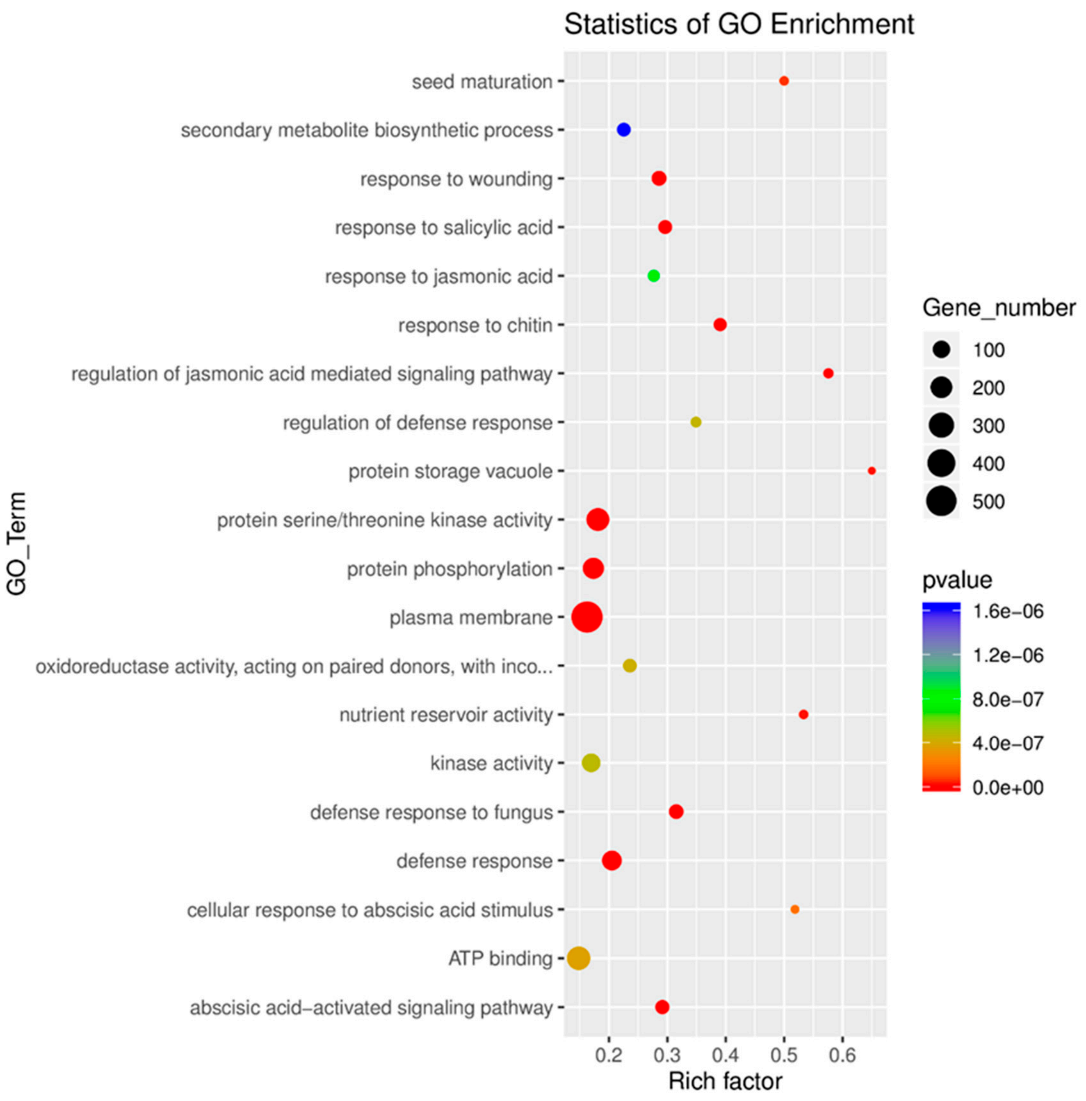

Statistics of GO Enrichment
seed maturation
secondary metabolite biosynthetic process
response to wounding
response to salicylic acid
response to jasmonic acid
response to chitin
regulation of jasmonic acid mediated signaling pathway
regulation of defense response
protein storage vacuole
protein serine/threonine kinase activity
protein phosphorylation
plasma membrane
oxidoreductase activity, acting on paired donors, with inco...
nutrient reservoir activity
kinase activity
defense response to fungus
defense response
cellular response to abscisic acid stimulus
ATP binding
abscisic acid–activated signaling pathway
GO_Term
0.2
0.3
0.4
0.5
0.6
Rich factor
Gene_number
100
200
300
400
500
pvalue
1.6e–06
1.2e–06
8.0e–07
4.0e–07
0.0e+00

D

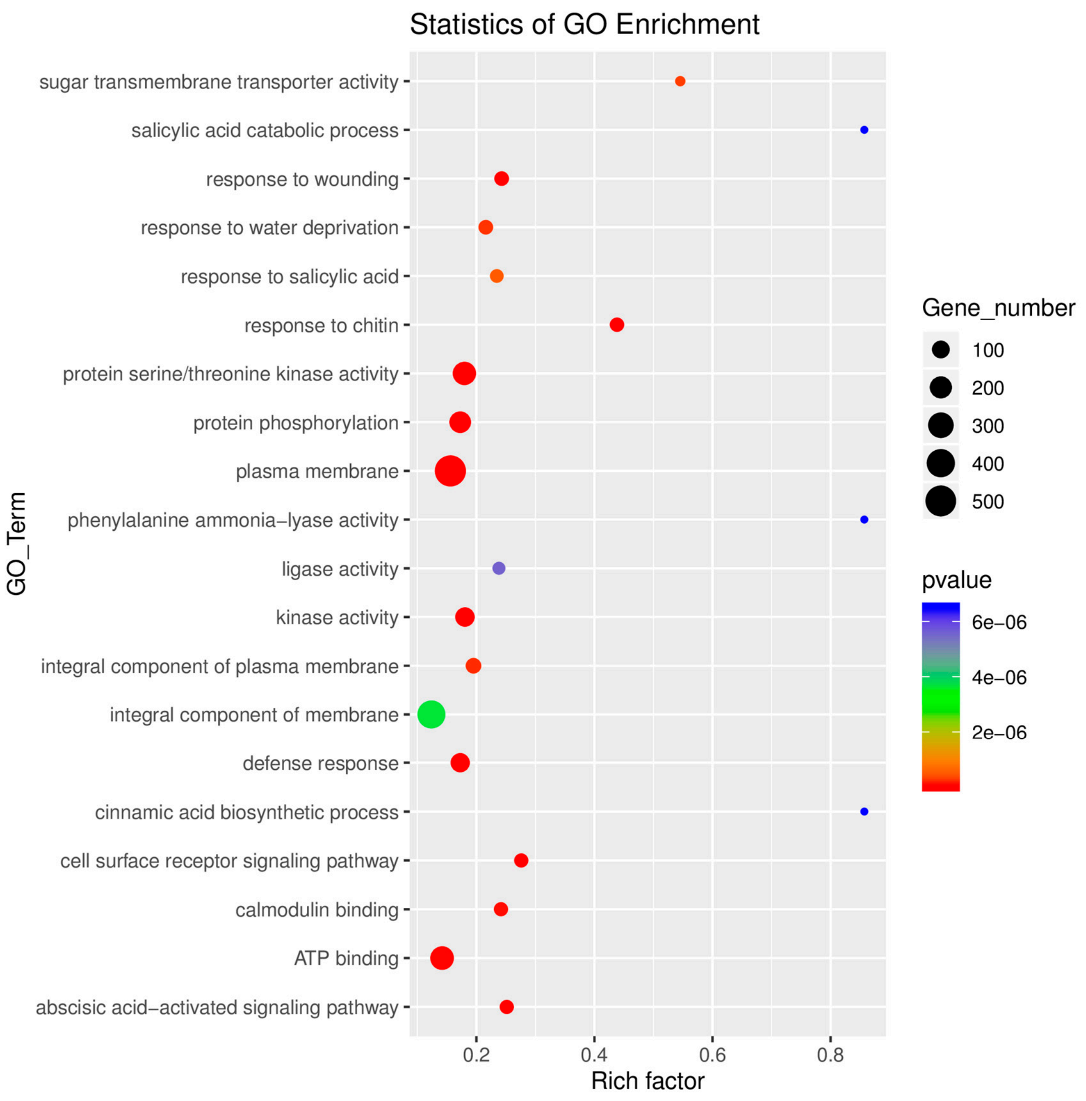

Statistics of GO Enrichment
sugar transmembrane transporter activity
salicylic acid catabolic process
response to wounding
response to water deprivation
response to salicylic acid
response to chitin
protein serine/threonine kinase activity
protein phosphorylation
plasma membrane
phenylalanine ammonia−lyase activity
ligase activity
kinase activity
integral component of plasma membrane
integral component of membrane
defense response
cinnamic acid biosynthetic process
cell surface receptor signaling pathway
calmodulin binding
ATP binding
abscisic acid−activated signaling pathway
GO_Term
0.2
0.4
0.6
0.8
Rich factor
Gene_number
100
200
300
400
500
pvalue
6e−06
4e−06
2e−06

Supplementary Figure S3 KEGG analysis of DEGs between WFYT025 in the environment of early rice. That showed the top 20 most represented categories and the number of transcripts predicted to belong to each category.

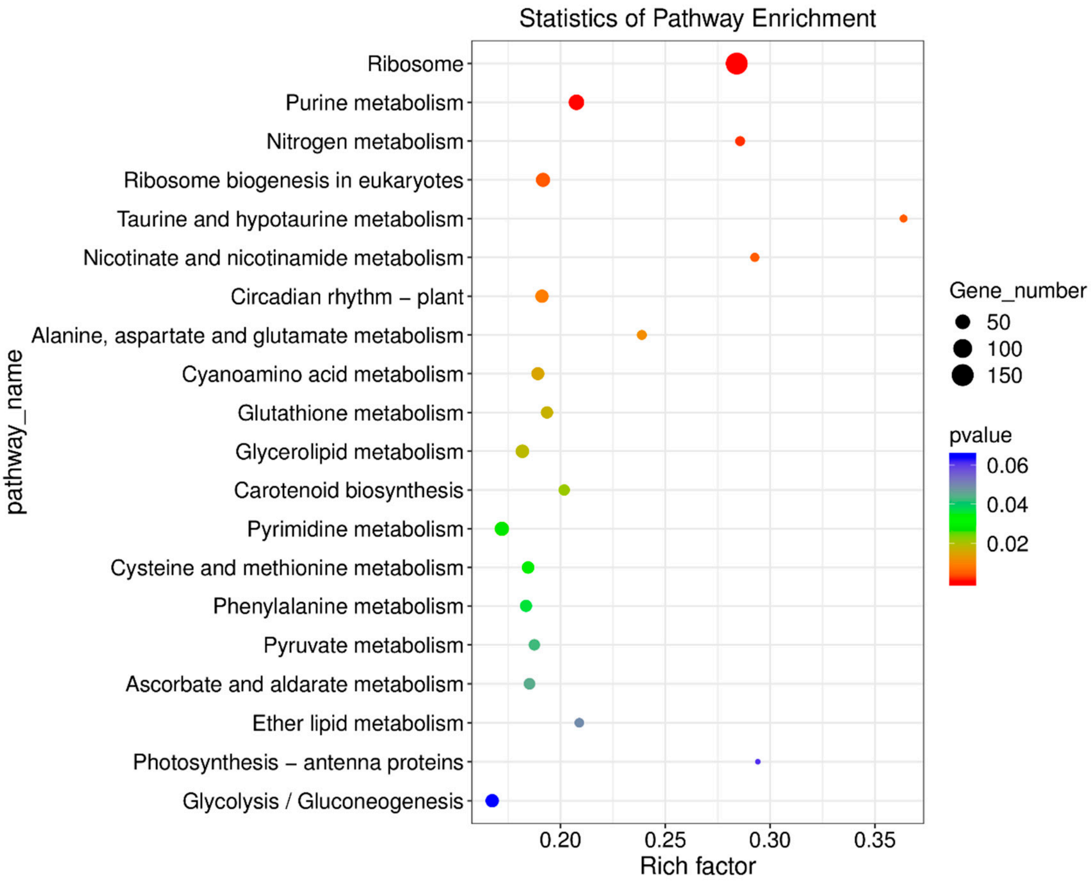

Supplementary Figure S4 Correlation between gene expression profile of co-expression module and three phenotypic data (TGW; Length; Width). The legend on the right shows the correlation coefficients, with positive correlation in red and negative correlation in blue. Since the aim is to find genes related to seed development, the focus is on the co-expression module with high correlation with TGW (module color is midnightblue, the gene expression profile in this module is moderately negatively correlated with the TGW phenotype)

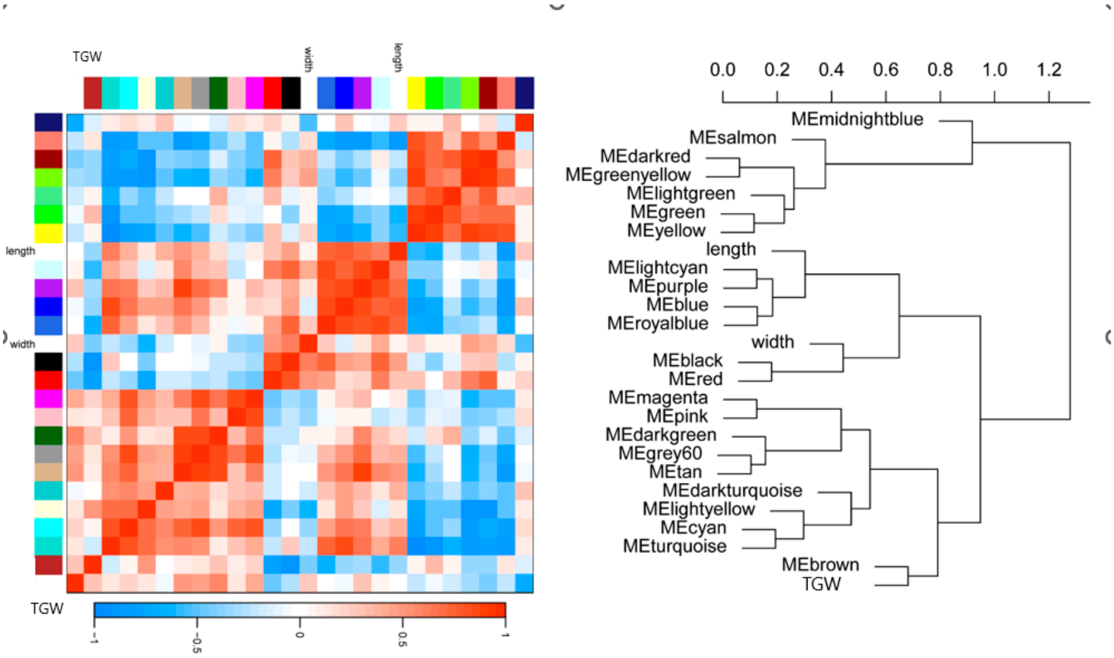

Supplementary Figure S5 Is the overall gene expression profile of the co-expression module MidnightBlue, which has the highest correlation with TGW.

There are 106 genes in this co-expression module. The graph is divided into three parts, the upper part is the expression heat map of the genes in the co-expression module. The middle part shows the relative expression of the eigengene in the module. The lower part shows the TGW phenotype values of the samples.

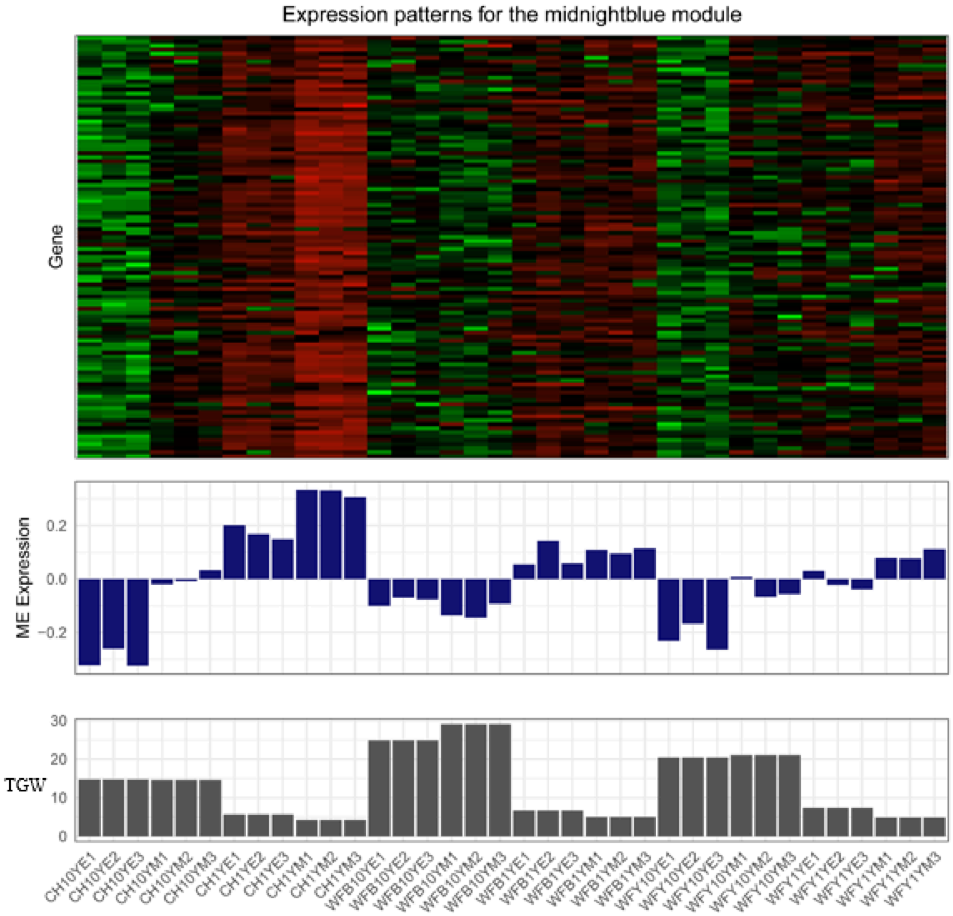

Supplement: Supplementary file 1 [file plants-12-02496-s001.zip › plants-2415875-supplementary.pdf]
